# Association between the colorectal cancer screening status of primary care physicians and their patients: Evidence from the Swiss Sentinella practice-based research network

**DOI:** 10.1016/j.pmedr.2023.102140

**Published:** 2023-02-13

**Authors:** Tamara Scharf, Claudia Hügli, Yonas Martin, Kali Tal, Nikola Biller-Andorno, Charles Dvořák, Jean-Luc Bulliard, Cyril Ducros, Kevin Selby, Reto Auer

**Affiliations:** a Institute of Primary Health Care (BIHAM), University of Bern, Switzerland; b Department of Infectious Diseases, Inselspital, Bern University Hospital, University of Bern, Bern, Switzerland; c Institute for Biomedical Ethics and History of Medicine, UZH, Zürich, Switzerland; d Past President Sentinella Network, Switzerland; e Center for Primary Care and Public Health (Unisanté), University of Lausanne, Switzerland

**Keywords:** PCP, primary care physicians, CRC, colorectal cancer, FOBT, fecal occult blood test, FIT, fecal immunochemical test, FOPH, Federal Office of Public Health, Primary health care, Screening, Colorectal cancer, Fecal occult blood testing, Colonoscopy, Variation in care

## Abstract

Swiss health insurance reimburses screening for colorectal cancer (CRC) with either colonoscopy or fecal occult blood test (FOBT). Studies have documented the association between a physician’s personal preventive health practices and the practices they recommend to their patients. We explored the association between CRC testing status of primary care physicians (PCP) and the testing rate among their patients.

From May 2017 to September 2017, we invited 129 PCP who belonged to the Swiss Sentinella Network to disclose their CRC test status and whether they had been tested with colonoscopy or FOBT/other methods. Each participating PCP collected demographic data and CRC testing status from 40 consecutive 50- to 75-year-old patients.

We analyzed data from 69 (54%) PCP 50 years or older and 2623 patients. Most PCP were men (81%); 75% were tested for CRC (67% with colonoscopy and 9% with FOBT). Mean patient age was 63; 50% were women; 43% had been tested for CRC (38%, 1000/2623 with colonoscopy and 5%, 131/2623, with FOBT or other non-endoscopic test). In multivariate adjusted regression models that clustered patients by PCP, the proportion of patients tested for CRC was higher among PCP tested for CRC than among PCP not tested (47% vs 32%; OR 1.97; 95% CI 1.36 to 2.85).

Since PCP CRC testing status is associated with their patients CRC testing rates, it informs future interventions that will alert PCPs to the influence of their health decisions and motivate them to further incorporate the values and preferences of their patients in their practice.

## Highlights

• Swiss health insurance reimburses CRC screening with colonoscopy or FOBT.
• Screening rates could rise if influence of PCP own screening choice was understood.
• Screened PCP had more patients screened for CRC compared to unscreened PCPs.

## 1 Introduction

Colorectal cancer (CRC) is the third most common cause of cancer mortality in high-income countries. (Bray et al., 2018) The lifetime risk of dying of CRC in Switzerland is 1.8% for men and 1.2% for women, (Federal Statistical Office NAfCR, 2021) but screening can cut this risk significantly. (Zauber, 2015, Lauby-Secretan et al., 2018) Swiss insurance reimburses CRC screening for average-risk patients between 50 and 69 years with either fecal immunochemical test (FIT) every-two years or colonoscopy every 10 years.

Though up to 70% of patients would agree to CRC screening, divided equally between those who would choose colonoscopy and FOBT, we previously found that the overall CRC testing rate in primary care in Switzerland was up to 48% and that most patients are tested with colonoscopy. (Martin et al., 2019) Patients’ decisions about CRC screening are influenced by their physician’s recommendation. (Beydoun and Beydoun, 2008) For example, in a telephone survey of 405 American patients aged 50 or older, those who received recommendations for FOBT from a clinician or nurse were almost four times more likely to have chosen that testing method. (Cibula and Morrow, 2003) If patients in Switzerland received more recommendations for FOBT, or if both methods were presented as good alternatives and patient values and preferences are given more room, this could raise screening rates in Switzerland (Inadomi et al., 2021).

PCP recommendations for CRC screening are influenced by their own screening choices. Physicians screened for CRC were more likely to recommend screening to their patients, based on an analysis of claims data drawn from a large health maintenance organization in Israel where both patients and physicians were insured. (Frank et al., 2013) Other studies found associations between physician’s personal preventive health practices, e.g., smoking cessation, vaccination, and screening, and the preventive health practices they recommended to their patients. (Frank et al., 2013, Frank et al., 2010, Frank et al., 2003,).

We hypothesized that the patients of PCP who had been screened for CRC screening would be screened at a higher rate than patients of PCP who had not been screened. Since there was little data on CRC health practices among primary care physicians (PCP) in Switzerland, we decided to collect data from PCP in the Swiss Sentinella network to test the association between a PCP’s self-reported CRC screening status and their patients’ screening status.

## 2 Methods

### 2.1 Population

We invited PCP from the Swiss Sentinel Surveillance (Sentinella) network to take a survey and fill in a structured form while collecting data from 40 consecutive non-urgent consultations with 50- to 75-year-old patients. Practicing PCP regularly fill out questionnaires within Sentinella, a cooperative surveillance project of the Federal Office of Public Health (FOPH) that is designed to monitor infectious diseases in the population. We have already used the same Sentinella data to report CRC screening rates among the same patients of participating PCP and on patient-level and PCP-level factors associated with CRC testing. (Braun et al., 2019, Martin et al., 2019).

### 2.2 Data collection

Between May and September 2017, we worked with the FOPH who invited 129 PCP from the Sentinella network to participate via mail. The mailed package included a brief overview of the project, printed survey and data collection forms (see Appendix). The FOPH provided us with demographic data on PCP from the Sentinella network, including sex, age, language region, practice location, geographic area of residency and population density, accessibility criteria (urban, intermediate, rural). (Federal Statistics Office, 2017).

PCP reported their screening status and if they generally recommend CRC screening to asymptomatic patients at average risk for CRC. They reported on this via printed mail questionnaires to the FOPH, who after anonymization forwarded the data to us.

We asked each PCP to collect CRC screening data on 40 consecutive patients between 50 and 75 years old who consulted for a non-urgent medical issue and participated in a face-to-face consultation over 5  min. PCP either include 40 eligible patients who met study requirements by recording their data in strict consecutive order or by including the first two eligible patients per half-day of work. PCP relied on a strict algorithm to record patient data on the collection form (Appendix Fig. 1). The PCP recorded the patient’s year of birth, sex, screening status, and medical factors that informed their decision to recommend or not screening/testing. PCP checked and recorded patients’ previous CRC screening status, either by consulting their records or asking the patient during the consultation. PCP could code status as FOBT/FIT more or less than two years ago, colonoscopy more or<10 years ago, other CRC screening tests, no CRC screening test, or screening status unknown. We did not distinguish between diagnostic and CRC screening tests in the data collection form. If the patient had been tested with both FOBT/FIT and colonoscopy, we considered only the colonoscopy. To simplify data collection, the form did not differentiate between guaiac-based (gFOBT) and immunological FOBT (iFOBT or FIT). If patients were seen more than once during the data collection period, we excluded the repeat visit. Patient data was irreversibly anonymized.

### 2.3 Primary outcome.

Our primary PCP-level outcome was self-reported previous CRC testing (colonoscopy and/or FOBT). Our primary patient-level outcome was previous CRC testing within the recommended interval (colonoscopy < 10 years or FOBT < 2 years), as reported by the PCP on the data collection form.

### 2.4 Statistical analyses.

We used descriptive statistics to report data on participating PCP and their patients. We calculated overall proportions of a) PCP tested for CRC and the chosen testing method and b) patients tested for CRC and the chosen testing method. We explored the association between a) the PCP’s own CRC testing status and b) that of their patients by using hierarchical multivariate mixed-effects logistic regression models that clustered patients by PCP and modeled a random effect by PCP. Fixed effects were modeled for patient characteristics (age and sex) and PCP characteristics (age, sex, language region, practice location).

We conducted our analyses with Stata version 15 (StataCorp LP, College Station, TX, USA).

The ethics committee of the canton of Bern waived approval for this study. Because we used a double, irreversible patient data anonymization process, the Swiss Human Research Act (REQ-2017–00280) did not apply to this project.

## 3 Results

Of 129 invited PCP, 91 responded to the invitation and provided data on their own CRC testing status and that of their patients. Of those, 70 PCP (54%) were eligible because they were 50 or older. We excluded 1 PCP because of unknown screening status. Our analysis included 69 PCP (54%) who collected data on 2,732 patients. We then excluded 109 patients aged under 50 or over 74, leaving 2,623 patients for analysis.

### 3.1 Baseline descriptive characteristics.

Most PCP were men (81%), aged 60 and older (61%), and worked in urban regions (71%). (Table 1). Mean age of the 2,623 patients was 63; half of the patients were women (Table 1).

**Table 1:** Description of characteristics of participating PCP aged 50–75 and consecutive patients aged 50–75 included by PCP.

	N (col%)	Up to date with CRC screening, N (%)	Colonoscopy within last 10 years. N (%)	FOBT within last 2 years-* N (%)
PCP characteristics	69	52 (75)	46 (67)	6 (9)
Gender				
Men	56 (81)	42 (75)	36 (64)	6 (11)
Women	13 (19)	10 (77)	10 (77)	0
Age				
50–59	27 (39)	21 (78)	19 (70)	2 (7)
>60	42 (61)	31 (74)	27 (64)	4 (10)
PCP working setting				
Alone	34 (49)	25 (74)	21 (62)	4 (12)
In a team	35 (51)	27 (77)	25 (71)	2 (6)
PCP practice location				
Urban	49 (71)	36 (74)	32 (65)	4 (8)
Intermediate	12 (17)	10 (83)	9 (75)	1 (8)
Rural	8 (12)	6 (75)	5 (63)	1 (13)
PCP language region				
German-speaking	45 (65)	35 (78)	31 (69)	4 (9)
French-speaking	19 (28)	14 (74)	12 (63)	2 (11)
Italian-speaking	5 (7)	3 (60)	3 (60)	0
Patient’s characteristics	2623	1131 (43)	1000 (38)	131 (5)
Gender				
Men	1300 (50)	534 (41)	469 (36)	65 (5)
Women	1323 (50)	597 (45)	531 (40)	66 (5)
*Age (only 50*–*75 included)*				
50–59	986 (38)	313 (32)	288 (29)	25 (3)
60–69	1020 (39)	473 (46)	420 (41)	53 (5)
70–75	617 (24)	345 (56)	292 (47)	53 (9)

PCP = primary care provider, CRC = colorectal cancer, FOBT = fecal occult blood test.

*FOBT or other non-endoscopic CRC screening test (for example blood test).

Disclaimer: Total of percentages can be slightly inaccurate, this is caused by rounding the numbers up or down.

### 3.2 CRC testing rates among PCP and their patients.

Among PCP, 75% (52 of a total of 69 reported they had been tested for CRC within recommended intervals (75% among men, 77% among women); 67% (46/69) with colonoscopy and 9% (6/69) with FOBT or another test (one with gFOBT, four with FIT and one with blood test). Of men PCP, 75% reported they had been tested; 77% of women PCP had been tested (Table 1). No woman PCP reported previous testing with FOBT.

Of the patients, 43% (1,131/2,623) had been tested for CRC within the recommended interval; 38% (1000/2623) with colonoscopy and 5% (131/2623) with FOBT. CRC testing rate among women was 45% and among men 41%. Testing rate increased with age from 32% among those aged 50–59 to 56% among those aged 70–75. (Table 1).

All but one PCP would recommend CRC screening to asymptomatic, average risk patients. The PCP who did not recommend screening had not undergone screening himself. Out of 40 consecutive patients, he reported 19 (48%) had been tested, all with colonoscopy.

### 3.3 Association between PCP CRC screening status and their patients.

The proportion of patients tested for CRC was higher among PCP tested for CRC (47% vs 32%). In both our univariate and multivariate adjusted models, CRC testing status of PCP was the only predictor significantly associated with a patient’s CRC testing status (multivariate adjusted odds ratio was 1.97; 95% CI 1.36 to 2.85) (Table 2).

**Table 2:** Association between covariates on the PCP level and CRC screening status and type of testing of consecutive patients included by PCP.

	P PCP	N Patients	Patients up to date with CRC screening. N (%)	Unadjusted odds Ratio* (95% CI)	Adjusted Odds Ratio** (95% CI)
PCP screening status					
PCP up to date with CRC screening	52 (75)	1968 (75)	920 (47)	1.97 (1.37–2.85)	1.97 (1.36–2.85)
PCP not up-to date with CRC screening	17 (25)	655 (25)	211 (32)	Ref	Ref
PCP					
Women	13 (19)	489 (19)	216 (44)	1.00 (0.63–1.52)	1.00 (0.64–1.56)
Men	56 (81)	2134 (81)	915 (43)	Ref.	Ref.
PCP characteristics					
Alone	34 (49)	1295 (49)	500 (39)	1.31 (0.95–1.80)	1.28 (0.93–1.77)
In a team	35 (51)	1288 (49)	627 (49)	Ref.	Ref.
PCP practice location					
Urban	49 (71)	1865 (71)	825 (44)	0.87 (0.68–1.12)	0.87 (0.67–1.11)
Intermediate	12 (17)	456 (17)	209 (46)	Ref.	Ref.
Rural	8 (12)	302 (12)	97 (32)	Ref.	Ref.
PCP language region					
German-speaking	45 (65)	1705 (65)	708 (42)	1.17 (0.89–1.54)	1.13 (0.86–1.50)
French-speaking	19 (28)	727 (28)	331 (46)	Ref	Ref
Italian-speaking	5 (7)	191 (7)	92 (48)	Ref	Ref

Abbreviations: CI: confidence interval. CRC: colorectal cancer, PCP: primary care provider,

* Unadjusted mixed-effects logistic regression models with each PCP modelled as a random effect to explore the association between PCP characteristics and the proportion of patients who were up to date with CRC screening.

** Results from multivariate mixed-effects logistic regression models with PCP characteristics modelled as a random effect to explore the association between PCP characteristics and the proportion of patients who were up to date with CRC screening. Models adjusted for PCP CRC screening status, PCP’ demographics (age, sex), PCP working place, practice location and language region. Models further adjusted for patients’ demographics (age, sex).

Disclaimer: Total of percentages can be slightly smaller or bigger than 100%, this is caused by rounding the numbers up or down.

## 4 Discussion.

We identified an association between PCP and their patients screening status and screening methods. The patients of PCP who had been tested for CRC were more likely to have been tested for CRC than patients whose PCP had not been tested (47% vs 32%). The PCP who participated in our study were more likely to have been tested within recommended intervals than their patients (75% vs 43%). Both PCP and patients were more likely to have been tested with colonoscopy than with FOBT. Patients were far more likely to be screened with colonoscopy, regardless of their PCP’s choice of screening method (46% vs 8% for PCP screened by colonoscopy; 31% vs 21.5% for FOBT). All PCP screened with FOBT (9%, 6/69) prescribed FOBT at least once during the data collection period. All except one PCP stated that they would recommend CRC screening to patients without CRC symptoms and average risk.

We found that PCP CRC screening status, may influence whether a patient is screened. In line with other studies, we found PCP not screened for CRC had a lower percentage of patients who were screened. (Frank et al., 2013) PCP who practiced in rural areas, worked in Italian-speaking regions of Switzerland, were over 60, and were male were more likely to be screened with FOBT. (Braun et al., 2019, Schneider et al., 2022) Like other studies, we found colonoscopy was the preferred screening method for both patients and PCP. (Hilsden et al., 2005).

We found all but one PCP stated they recommend CRC screening to asymptomatic, average risk patients. Previous studies found only 25% of PCP recommending screening in regular consultations (Triantafillidis et al., 2017). Since we relied on data reporting previous testing conducted over the last 10 years for colonoscopy, we can’t track whether PCP actually recommended CRC screening to their patients in the past.

Some of our results differ from previous studies. We found a higher rate of PCP screened for CRC (75%) compared to a Canadian study (61% of eligible PCP were screened for CRC), perhaps because Sentinella PCP are more sensitized to screening or because screening rates of PCP are higher in the Swiss population (Frank et al., 2013).

CRC screening rates among patients were lower than the 65% recommended by European guidelines, albeit in line with participation level in the first CRC screening program in Switzerland, and patients were screened mostly by colonoscopy (Brändle and Bulliard, 2022). Our results suggest the predominance of colonoscopy may largely be explained by physician preference (Martin et al., 2019). Evidence from a randomized controlled trial testing the effect of choice on tests performed suggests that patients appear as likely to choose FOBT as colonoscopy and if choice was given, patients followed through with CRC screening more often compared to patients who had been offered colonoscopy alone (Inadomi et al., 2021). Offering only colonoscopy might partially explain why screening rates are lower than recommended. Along with other interventions, encouraging PCPs to offer both methods could allow more patients to choose the test that matches their preferences and values and increase CRC screening rates overall (Martin et al., 2019).

## 5 Limitations

The PCP who participated in our study belong to a network that regularly gathers health monitoring data. They may be more familiar with cancer screening and public health guidelines than their peers and more motivated to choose to be screened. The screening rate among PCP and their patients might overestimate the true CRC screening rate among the general population of PCP.

Because we used de-identified data and relied on PCP self-reports, we could not verify the CRC testing rates of physicians and of their patients. Reliance on memory of PCP may have caused us to overestimate patient screening rates. However, our estimated CRC testing rates concurred with our previous estimates for the general Swiss population based on results from a nationwide health survey conducted in 2017 and claims data analyses from a large Swiss health insurance. (Schneider et al., 2022, Bissig et al., 2022).

## 6 Conclusion

Since PCP CRC testing status is associated with the CRC testing rates of their patients, it informs future interventions that will alert PCPs to the influence of their own health decisions and motivate them to further incorporate the values and preferences of their patients in their daily practice.

## 7 Sources of funding

This work was supported by the Swiss National Science Foundation, National Research Program 74 (NFP74 407440-167519) “Smarter Health Care” and the Swiss Cancer Research Foundation, Health Services Research (HSR-4366-11-2017). The funders had no role in the design or conduct of the study, in the collection, management, analysis, or interpretation of data, or in the preparation, review, or approval of the manuscript. None of the authors report a conflict of interest.

## Declaration of Competing Interest

The authors declare that they have no known competing financial interests or personal relationships that could have appeared to influence the work reported in this paper.

## Figures and Tables

**Fig. 1:**
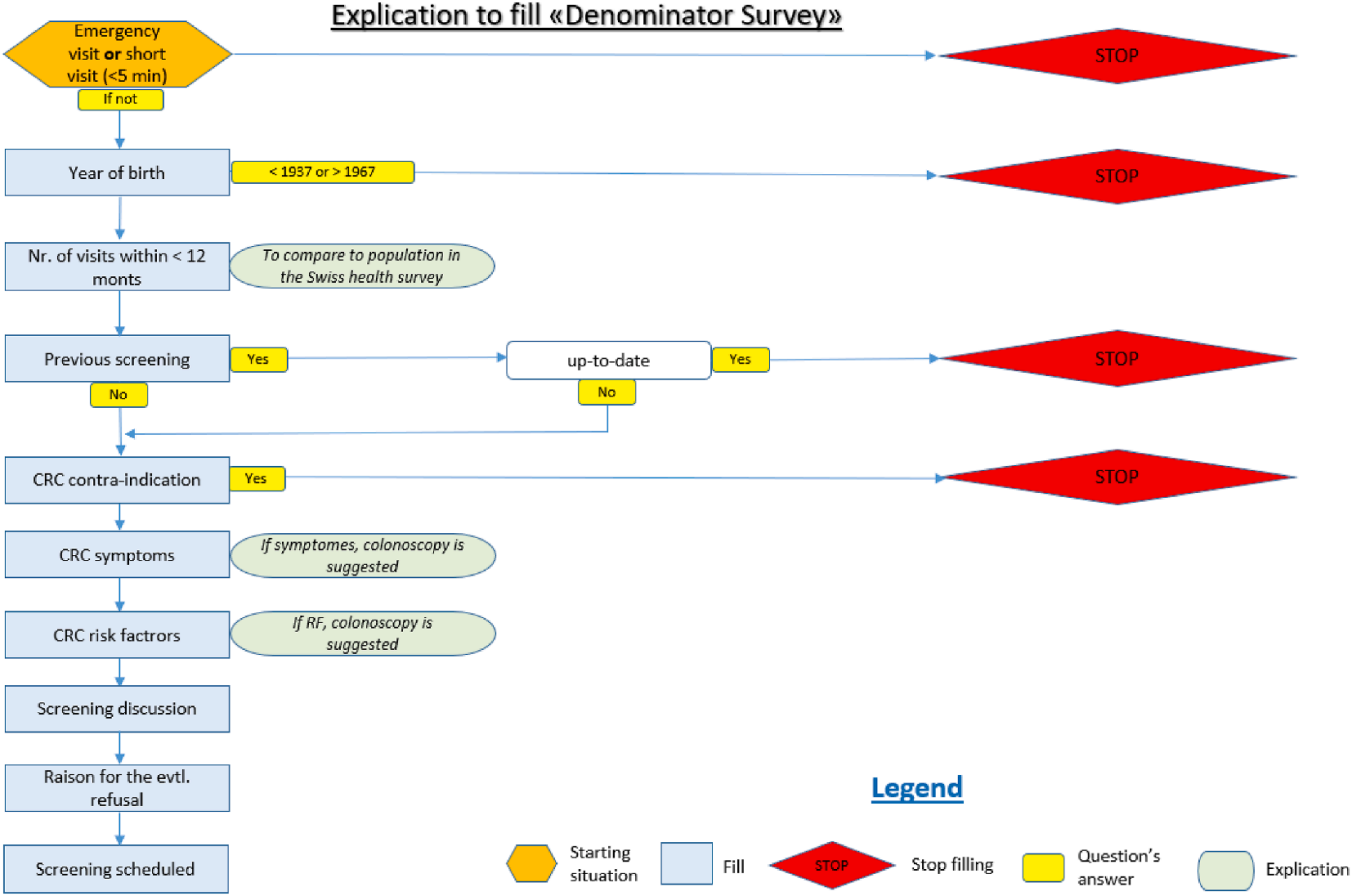
Flow chart data collecting.

**Figure:**
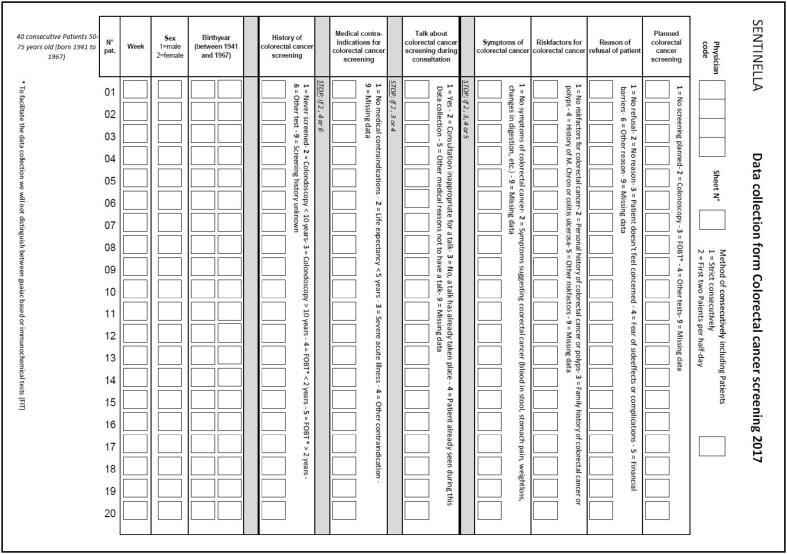


## Data Availability

Data will be made available on request.

## Appendix

Collection form:
